# Exploring Current Perspectives on a Doctor of Philosophy Degree in Genetic Counseling

**DOI:** 10.1002/jgc4.70276

**Published:** 2026-08-17

**Authors:** Elizabeth Coffran, Tiffiney R. Hartman, Anna Madden, Ashika Mani, Laura Conway, Kathleen D. Valverde

**Affiliations:** ^1^ Master of Science in Genetic Counseling Program Perelman School of Medicine, University of Pennsylvania Philadelphia Pennsylvania USA; ^2^ GeneDX Gaithersburg Maryland USA; ^3^ Center for Clinical Epidemiology and Biostatistics, Biostatistics Analysis Center Perelman School of Medicine, University of Pennsylvania Philadelphia Pennsylvania USA

**Keywords:** doctoral degree, education, genetic counseling, professional development

## Abstract

Since the profession's inception, the entry‐level and terminal degree for genetic counselors (GCs) has remained a master's degree. Interest in a PhD in Genetic Counseling has been explored previously; however, no studies on this topic have been published since 2015. Given the continued growth in the number of genetic counselors and the expansion of their roles, this study investigated current perceptions of GCs regarding the creation of a PhD in Genetic Counseling. A 56‐question, investigator‐developed survey collected demographic information, assessed participants' personal interest in a PhD in Genetic Counseling, and included Likert scale questions exploring perceptions of how such a degree might affect the profession, as well as factors influencing participants' decisions to pursue it. A final open‐ended question allowed participants to elaborate on their responses. Participants (*n* = 514) had an average of 9.5 years of experience post‐master's degree. Most (69.1%) supported the development of a PhD for GCs with several years of experience, while a smaller proportion (57.3%) supported it for recent graduates. Overall, 39.6% had considered earning a PhD, and 37.4% indicated interest in pursuing a PhD in Genetic Counseling. Factors most influencing their decision included time commitment and potential salary increase. Perceived benefits included enhancement of research skills (85.9%) and increased access to faculty positions (83.1%). About half (48.6%) indicated that a PhD in Genetic Counseling could reduce opportunities for GCs with a master's degree. Open‐ended comments indicated confusion regarding the distinction between a PhD and other advanced degrees, such as a clinical doctorate. Overall, while many participants supported the development of a PhD in Genetic Counseling, this support remained mixed and tempered by concerns about its impact on master's‐trained counselors. These findings suggest that further exploration of the role and implications of a PhD in Genetic Counseling is warranted.

## Abstract (highlights)

**What Is Known About This Topic?:**

Discussion surrounding the development of a PhD for GCs beyond the entry‐level master's degree has been ongoing for over three decades. However, no studies on this topic have been published in the past 10 years, despite significant advancements in the field and expansion beyond the clinic to encompass more research, laboratory, and academic roles.

**What Does This Paper Add to the Topic?:**

This study provides insights into GCs' attitudes toward pursuing a PhD, demonstrating support for the degree alongside concerns about cost, accessibility, and potential impacts on those with a terminal master's degree. These findings highlight the need for further evaluation of how a PhD in Genetic Counseling may influence research training, career development, and equity within the field, as well as the need for careful consideration of program design should the degree become more widely implemented.

## 1 Introduction

For more than 30 years, genetic counselors (GCs) have been discussing whether to develop advanced degrees beyond the entry‐level master's degree (Scott et al. 1988). A doctorate, or doctoral degree, is an umbrella term for terminal degrees that include academic degrees such as a PhD or an EdD, as well as professional degrees such as an MD, PsyD, or JD. A PhD (Doctor of Philosophy) is a research degree focused on specific disciplines such as education or science. In September 1989, the National Society of Genetic Counselors (NSGC) Educational Committee convened the 1989 Asilomar Meeting on Education in Genetic Counseling with 35 attendees representing 15 training programs for GCs, five training programs for genetic nurse specialists, and others with interest in GC education, to discuss topics including potential advanced graduate education options in genetic counseling. That same month, a survey was distributed to all 565 members of the NSGC that found that 53.4% (*n* = 180/337) of respondents identified a need for a doctoral degree in Genetic Counseling, citing anticipated benefits such as improved professional recognition, opportunities for specialization, increased knowledge, and higher compenzation (Gaupman et al. 1991). Despite clear interest among GCs at the time and several positive outcomes that could result from the creation of a doctoral degree identified during the meeting, participants failed to reach conclusions on the need or advisability of establishing a doctorate in Genetic Counseling, and ultimately recommended deferring decisions until key areas of uncertainty were addressed (Walker et al. 1990).

Subsequent work has explored potential roles for doctoral‐trained GCs. Wallace and colleagues interviewed 30 employers of GCs and found that most (23/30) envisioned potential roles for PhD‐trained GCs, particularly in academic and research settings, public health initiatives, and personalized medicine (Wallace et al. 2008). These findings suggested an employment niche for individuals with a PhD in Genetic Counseling, as these roles were perceived to complement rather than compete with those of master's‐trained GCs (Wallace et al. 2008).

Despite decades of interest, uptake of doctoral training among GCs has remained low. Since 2000, when NSGC began collecting data through the Professional Status Survey (PSS), the proportion of GCs holding a doctoral degree in any field has remained stable at approximately 2%–3% (Marra et al. 2023; NSGC PSS 2000– 2024). In the most recently published PSS in 2024, < 1.0% (*n* ≤ 5) of GCs reported having an MD, and only 2.8% of GCs (*n* = 72/2615) reported having a PhD, EdD, DrPH, ScD, or PhilD (National Society of Genetic Counselors 2024). Notably, these data do not specify the field of study or whether respondents first earned a master's degree in genetic counseling or a PhD in another discipline. It is unclear whether this longstanding stability reflects the absence of a defined pathway for advanced training in genetic counseling or other contributing factors (Marra et al. 2023).

To explore the impact of doctoral training on the field, Atzinger and colleagues interviewed 31 GCs with doctoral degrees in various fields (Atzinger et al. 2007). While some respondents (25.8%) did not think their practice differed from master's‐trained GCs, others reported increased involvement in research (22.6%), greater autonomy (12.9%), and reduced patient contact (6.5%). Most respondents (83.9%) believed that a doctoral degree, specifically in genetic counseling, would have a positive impact, though some noted that its value would depend on the curriculum. Others were concerned that it would minimize opportunities for master's‐level GCs or suggested that it would be of no benefit for clinical GCs. Currently, over 35 years since the Asilomar Meeting, only two PhD programs in Genetic Counseling exist worldwide, one with enrolled participants at the University of Technology in Sydney, Australia (“Doctor of Philosophy [PhD Thesis: Genetic Counseling]” uts.edu.au/courses/doctor‐of‐philosophy‐phd‐thesis‐genetic‐counseling), and the other that will matriculate the first class in the fall of 2027 at the University of British Columbia (“Doctor of Philosophy in Medical Genetics, Sub‐Specialization in Genetic and Genomic Counseling [PhD]” grad.ubc.ca/prospective‐students/graduate‐degree‐programs/phd‐medical‐genetics‐genetic‐genomic‐counseling).

An alternative to a PhD is a Clinical Doctorate (ClinD), which is focused on advanced clinical training for professional practice and direct patient care. Some allied health professions, such as physical therapy and occupational therapy, have developed a ClinD in response to a perceived need for additional professional training for practicing clinicians. These programs often incorporate structured research components to promote evidence‐based practice, compared to a PhD's focus on original scholarly research (“Accreditation Standards.” n.d., https://acoteonline.org/accreditation‐explained/standards/). A similar model has been considered for genetic counseling, with representatives of the then 31 accredited genetic counseling programs voting at a retreat in 2012 to retain the master's as the entry level and terminal degree and to establish a task force to evaluate the role for advanced training for certified GCs (Reiser et al. 2015). The Genetic Counseling Advanced Degree Task Force (GCADTF) considered votes cast by members of various genetics organizations, in addition to a “vote” based on survey results assessing practicing GCs and students views about whether the profession should maintain the master's as the terminal degree or move toward the ClinD as the required entry level degree for new graduates. The survey results identified that most respondents were not in favor of moving to an entry level ClinD at the time (Nagy et al. 2015). The GCADTF ultimately recommended that the profession continue to explore opportunities for advanced training, given that the data showed that GCs are looking for increased opportunities for career advancement, despite consistent concerns about barriers and potential negative impacts on the profession.

Genetic counseling students receive research methodologies training and education, and until 2023, students were required to perform research and scholarly activities (“Standards of Accreditation for Graduate Programs in Genetic Counseling.” 2019, https://www.gceducation.org/standards‐faqs/). Updated standards require that students must “demonstrate knowledge of how GCs engage and contribute to the research process,” although many programs continue to require a formal thesis, independent research project, or capstone project (“Practice Based Competencies for Genetic Counselors”, 2023, https://www.gceducation.org/forms‐resources/). This research training has prepared many in the field to contribute to research as part of their professional roles. In the 2024 PSS, 19.0% (*n* = 483/2540) of GCs reported research as a role they perform, 60.1% (*n* = 1524/2534) reported participating in at least one research activity, and 37.8% (957/2534) had written or contributed to a manuscript (National Society of Genetic Counselors 2024). The impact of GCs on research is illustrated by 992 unique publications, including at least one GC as an author over four decades at a single major research institution (Muraresku et al. 2024). Due to the current limitation of a dedicated PhD in Genetic Counseling to only two institutions outside of the United States, those interested in advanced research training typically pursue advanced degrees in related fields. Notably, individuals with advanced degrees appear to be overrepresented among program leadership; an informal review of program websites conducted in 2022 found that 36 of 155 individuals (23.2%) held doctoral degrees, including 33 PhDs and 3 EdDs.

Since the publication of previous studies on GCs' attitudes toward a doctoral degree, the field has grown tremendously from approximately 230 students graduating in 2012, the year the data were collected for the GCADTF, to approximately 500–550 students graduating in 2024 (https://natmatch.com/gcadmissions/statistics.html). Considering this expansion, we surveyed GCs to reassess current interest in and attitudes toward a PhD in Genetic Counseling.

## 2 Methods

### 2.1 Study Design

This was a descriptive and exploratory survey designed to capture a current snapshot of GCs' opinions on the creation of a PhD in Genetic Counseling. Data was collected between November 2022 and January 2023. This project was determined to be exempt by the University of Pennsylvania Institutional Review Board (protocol #852004).

### 2.2 Participant Recruitment

The target population was board certified GCs. A REDcap (Research Electronic Data Capture) link to a 56‐question, investigator‐developed survey was distributed via the NSGC, the American Board of Genetic Counseling (ABGC), and the Pennsylvania Association of Genetic Counselors (PAGC). GCs who received the survey were asked to forward the email to colleagues to maximize participation. To be enrolled in the study, participants had to be able to read and respond to the survey in English.

### 2.3 Online Survey

Study data were collected and managed using REDCap electronic data capture tools hosted at the University of Pennsylvania. REDCap is a secure, web‐based software platform designed to support data capture for research studies (PA Harris et al. 2009). The online survey (Supplemental Section) was created by the research team, informed by past literature (Gaupman et al. 1991) and a prior unpublished exploratory study on the views of a clinical doctorate in genetic counseling. The development process involved iterative meetings among the study team and piloting the questionnaire with a small number of GCs at their institution to refine the content, clarify questions, and tailor the survey to appropriately gather participants' perspectives on the topic.

The survey collected demographic information, assessed interest in a PhD in Genetic Counseling, included Likert scale items evaluating the potential impact of a PhD on the field, and examined factors influencing participants' interest in pursuing a PhD. An additional open‐ended question invited participants to elaborate on their responses.

### 2.4 Data Analysis

A total of 514 individuals completed the survey and were included in this study. Of those, some did not answer every question, resulting in varying response numbers across items. Descriptive statistics were calculated based on the 514 total individuals, accounting for missing data. Statistical analyses were performed using Excel, SAS V9.4 statistical package (SAS Institute Inc., Cary, NC), and R Statistical Software (v4.1.2; R Core Team 2021). Analysis compared outcomes using the total number of responses for each question, not including missing data, resulting in avariable denominator number. Data were analyzed in aggregate and subgroups using parametric and nonparametric summary statistics (percentages, means, standard deviations, medians). Associations among variables were investigated using cross‐tabulations, 2 group tests (*t*‐test and/or Wilcoxon, depending on the variables distribution), and logistic regression. We used a *p* < 0.05 threshold for statistical significance, consistent with conventional standards in biomedical research.

Open‐ended responses regarding factors influencing individual decisions to pursue or not pursue an advanced degree and a prompt giving participants the opportunity to “provide any additional comments you may have” were analyzed by TRH, KDV, and AM using inductive content analysis (Cavanagh 1997; Coulston et al. 2025; Downe‐Wamboldt 1992; Graneheim and Lundman 2004; Krippendorf 2004; Vears and Gillam 2022). TRH and KDV independently reviewed the comments via an inductive process, identifying codes based on the actual content of the data set, then reviewed and collaboratively agreed upon the final content categories. KDV and AM then assigned each response to one or more content categories for final analysis.

## 3 Results

### 3.1 Demographics

Five hundred and fourteen participants consented to the study. A 2023 study estimated ~7100 GCs in North America, calculated from reports of 6517 certified counselors from the ABGC in March 2023 and 380 from the CBGC in February 2024 (Ormond et al. 2024). In April of 2021, the ABGC reported 5629 certified GCs (https://www.nsgc.org/Research‐and‐Publications/Professional‐Status‐Survey/Past‐Professional‐Status‐Surveys). Although it is impossible to determine the exact number of individuals who received the survey invitation in 2022, if 7100 GCs received the survey, the minimum response rate was 7.2% (514/7100). Most respondents were female (92.0%, *n* = 473/514), white (89.1%, *n* = 458/514), and worked at an academic hospital/medical facility (51.0%, *n* = 262/514) (Table 1). These proportions were comparable to those reported in the 2022 PSS (93% female, 89% white, and 45.0% working at an academic hospital), though those working at an academic hospital were overrepresented in our survey, and the PSS included some additional job settings not listed as options. The majority (71.0%) were 40 years old or under (similar to 69.0% of respondents under 40 years of age in the 2022 PSS), with an average age of 36 and a range of 23–73. Over half (64.4%) of respondents had less than or equal to 10 years of experience (61.0% in the 2022 PSS), with an average of 9.5 years and a range of 0–43 (Table 1). A minority (5.6%, *N* = 29/514) of respondents reported having a doctoral degree, which is higher than in the 2022 PSS (2.0%). Sixteen respondents (55.2%) earned their doctorate before, 12 (41.4%) after their genetic counseling master's degrees, and one did not respond. Interestingly, for those earning their doctorate before the master's, all reported a doctorate in the biomedical sciences: 62.5% (10/16) in molecular biology/genetics, 31.3% (5/16) in biology/biomedical science, and 6.3% (1/16) in chemistry. Those earning the PhD after the master's showed a shift toward public health (25%, 3/12), the humanities and society (16.7% 2/12), and education/administration (16.7%, 2/12), with fewer focusing on human or medical genetics (41.7%, 5/12). Many respondents (55.6%, *n* = 283/509) reported currently participating in research (50.0% in the 2022 NSGC PSS), which ranged from guiding students through thesis research to working full time in research as a principal investigator or co‐investigator on projects.

**TABLE 1:** Demographics of participants who answered and the percentage of totals for each category. Not all participants answered all questions. For age and years of experience, data were combined into 5‐year increments in the table.

	Overall (*n* = 514)
Gender	
Female	473 (92.0%)
Male	28 (5.4%)
Nonbinary	2 (0.4%)
Prefer not to say	5 (1%)
Missing	6 (1.2%)
Ancestry	
African American	9 (1.8%)
Asian	21 (4.1%)
Middle Eastern/North African	8 (1.6%)
White	458 (89.1%)
Missing	18 (3.5%)
Ethnicity	
Hispanic or Latinx	16 (3.1%)
Not Hispanic or Latinx	479 (93.2%)
Missing	19 (3.7%)
Age	
20–25	37 (7.2%)
26–30	148 (28.8%)
31–35	114 (22.2%)
36–40	66 (12.8%)
41–45	47 (9.1%)
46–50	35 (6.8%)
51+	58 (11.3%)
Missing	9 (1.8%)
Years of experience	
0–5	233 (45.3%)
6–10	98 (19.1%)
11–15	66 (12.8%)
16–20	41 (8.0%)
21–25	36 (7.0%)
26–30	18 (3.5%)
31+	17 (3.3%)
Missing	5 (1.0%)
Geographic region	
New England (CT, MA, ME, NH, RI, VT, CN, maritime provinces)	34 (6.6%)
Mid‐Atlantic (DC, DE, MD, NJ, NY, PA, VA, WV, PR, VI, Quebec)	138 (26.8%)
Southeastern (AL, FL, GA, KY, LA, MS, NC, SC, TN)	69 (13.4%)
Midwestern (AR, IA, IL, IN, KS, MI, MN, MO, NE, ND, OH, OK, SD, WI, Ontario)	139 (27.0%)
Mountain States (AZ, CO, MT, NM, TX, UT, WY, Alberta, Manitoba, Saskatchewan)	60 (11.7%)
Western (AK, CA, HI, ID, NV, OR, WA, British Columbia)	67 (13.0%)
Missing	7 (1.4%)
Job setting	
Academic hospital/medical facility	262 (51.0%)
Non‐academic hospital/medical facility	81 (15.8%)
Private practice or physician private practice	9 (1.8%)
Non‐profit or government organization	10 (1.9%)
Diagnostic Laboratory	88 (17.1%)
Other company	22 (4.3%)
University, college, or training program	27 (5.3%)
Other	11 (2.1%)
Missing	4 (0.8%)

### 3.2 Interests

Less than half of participants (39.6%, *n* = 190 of 480 who answered this question) indicated they had considered earning a doctorate since receiving their master's degree. Of these, 184/190 (96.8%) responded to the type of field they had considered, often with more than one field, totaling 245 possible fields. The fields of most interest include Clinical Genetics/Human Genetics/Genomics (31.5%, listed by 58/184 respondents), Education (20.1%, *n* = 38/184), Public Health (16.8%, *n* = 31/184), Counseling/Psychology (10.3%, *n* = 19/184), and Ethics (7.1%, *n* = 13/184). Eighteen out of 184 (9.8%) specified a ClinD or PhD in Genetic Counseling when answering this question. Responses to this question were quite varied with 23 distinct fields, including implementation science, epidemiology, medicine, behavioral science, bioinformatics, biostatistics, and genetic anthropology (all with fewer than ten respondents). 4.3% (*n* = 8/184) mentioned a PhD in their clinical subspecialty, such as cancer, cardiac, or neuroscience genetics. More than half of respondents indicated interest in working in research (57.1%, *n* = 290/508) or teaching/academia (76.6%, *n* = 389/508). Of note, 37.4% (*n* = 190/508) of participants answered yes to a question about their interest in pursuing a PhD in Genetic Counseling. There was no statistically significant difference in interest in pursuing this degree between men (28.6%) and women (35.4%) (*p* = 0.1505). When asked about whether they supported the idea of a PhD in Genetic Counseling for those with several years of experience or recent genetic counseling graduates, a higher percentage (69.1%, *n* = 347/502) supported the degree for GCs with several years of experience than for recent genetic counseling graduates (57.3%, *n* = 287/501). This difference is statistically significant (McNemar test *p* < 0.0001).

### 3.3 Perceptions of the Impact of a PhD on the Genetic Counseling Profession

**FIGURE 1:**
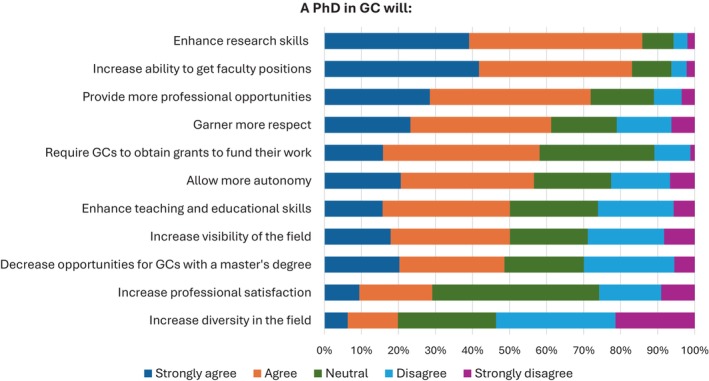
Participants were asked to indicate their agreement with the indicated statements pertaining to what a PhD in GC may do using a 5‐point Likert scale: Agree strongly, agree, neutral, disagree, and strongly disagree for each question. Not all participants answered all questions.

Participants were asked about how a PhD in Genetic Counseling might affect the field. The two most agreed upon impacts of a PhD would enhance GCs' research skills (85.9%, *n* = 437/509) and increase GCs' ability to get faculty positions (83.1%, *n* = 422/508, Figure 1). Half or more of the respondents agreed that a PhD in Genetic Counseling would have the following positive effects: providing GCs with more professional opportunities (71.9%, *n* = 366/509), garnering GCs more respect (61.2%, *n* = 311/508), allowing GCs to be more autonomous (56.6%, *n* = 288/509), enhancing GCs' teaching and educational skills (50.1%, *n* = 255/509), and increasing the visibility of the genetic counseling field (50.1%, *n* = 255/509). Many indicated concern about negative effects, specifically that a PhD in Genetic Counseling would require GCs to obtain grants to fund their work (58.1%, *n* = 294/506) and that a PhD would decrease opportunities for GCs with a master's degree (48.6%, *n* = 247/509). When asked about GCs with a leading role in research but not specifying individuals with a PhD, 46.6% of participants (*n* = 238/511) agreed that GCs who have a leading role in research have more autonomy than those who do not. Further analysis found that individuals currently involved in research were more likely to agree with this statement (55.6%, *n* = 283/509) compared to those not involved in research (44.4%, *n* = 226/509). This difference represents a statistically significant association between participation in research and the perception of increased autonomy for those in research (*p* < 0.0001).

### 3.4 Factors That Influence Interest in a PhD

**FIGURE 2:**
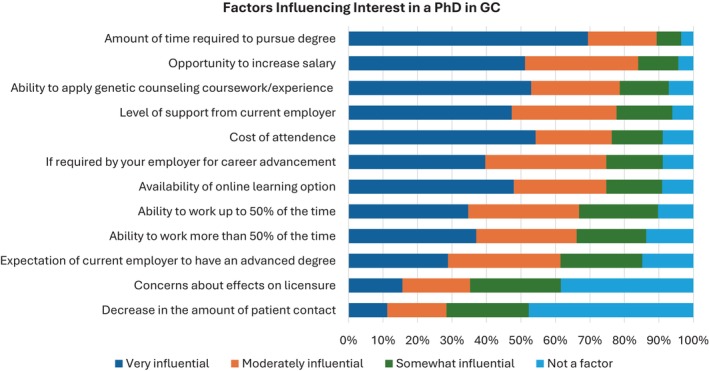
Participants were asked to indicate the extent to which the indicated factors would influence their decision to pursue a PhD in genetic counseling using a 4‐point Likert scale: Not a factor, somewhat influential, moderately influential, and very influential. Not all participants answered all questions.

**FIGURE 3:**
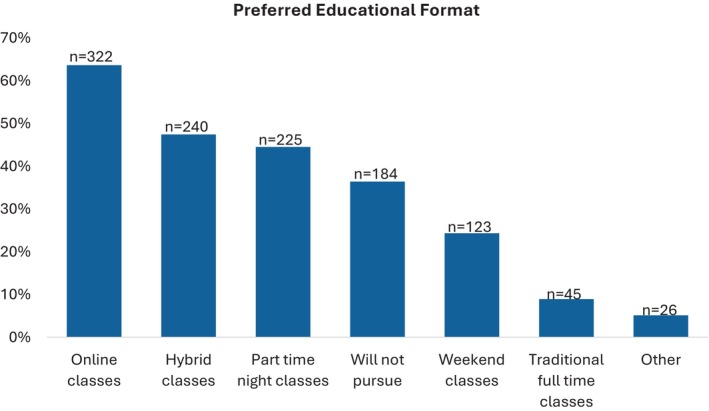
Study participants were asked which of the indicated options they would prefer if they were to pursue their doctorate in genetic counseling. Participants were instructed to select all options that apply. Five hundred six participants completed this question. The percentage of respondents who selected each option is shown by the bar graph with the number selecting that option indicated above the bar.

Aspects of a program that could influence the decision to return to school for a PhD in Genetic Counseling are illustrated in Figure 2. The most influential factors (based on those who answered moderately or very influential) were the time required to pursue the degree (89.3%, *n* = 453/507), opportunity to increase salary (84.0%, *n* = 425/506), ability to apply masters coursework and work experience as credit (78.7%, *n* = 398/506), and level of support from current employer (77.7%, *n* = 394/507). Cost of attendance was a concern for 76.3% (*n* = 387/507). Fewer than 35% of respondents indicated that a decrease in patient contact and concern about effects on licensure were either moderately or very influential. Regarding program structure, most participants preferred an online learning option (63.6%, *n* = 322/506), followed by part‐time evening classes (47.4%, *n* = 240/506). Almost half of the participants stated that the availability of an online learning option would be very influential in their decision to pursue a PhD. The least preferred class type by far was traditional full‐time classes (8.9%, *n* = 45/506). The preferred educational format is summarized in Figure 3.

Interest in the degree correlates with several factors. Currently participating in research (*p* < 0.001), having an interest in teaching/academia (*p* < 0.001), and working in an academic hospital/medical facility (*p* = 0.0029) significantly increased interest (Table 2). Having an interest in teaching/academia also positively influenced support for the degree, both among recent genetic counseling graduates (*p* = 0.0057) and GCs with several years of experience (*p* = 0.0021).

**TABLE 2:** 2‐sample *T*‐test used to compare interest in pursuing a PhD with responses from shown questions. Statistically significant comparisons shown.

Interest in pursuing a PhD in GC degree	No (*n* = 318)	Yes (*n* = 190)	*p*
Do you currently participate in research?			
No	159 (50.0%)	60 (34.7%)	< 0.001
Yes	156 (49.1%)	124 (65.3%)	
Missing	3 (0.9%)	0 (0%)	
Are you interested in working in teaching/academia?			
No	100 (31.4%)	19 (10.0)	< 0.001
Yes	215 (67.6%)	170 (89.5%)	
Missing	3 (0.9%)	1 (0.5%)	
Do you currently work in an academic hospital/medical facility?			
No	171 (53.8%)	76 (40.0%)	0.00285
Yes	145 (45.6%)	114 (60.0%)	
Missing	2 (0.6%)	0 (0.0%)	

**FIGURE 4:**
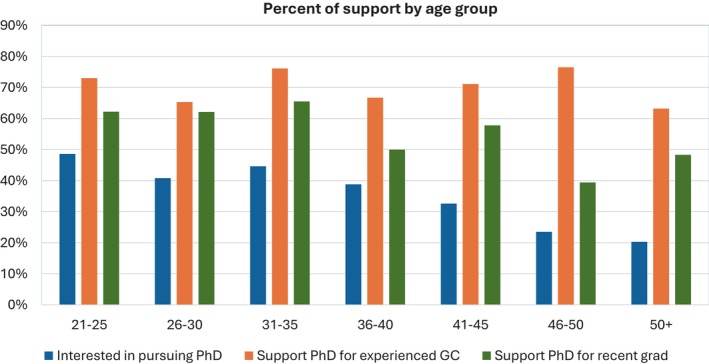
Percentage of participants interested in pursuing a PhD in GC, support of the degree for genetic counselors with several years of experience, and support of the degree for recent MSGC graduates broken down by age of respondent. The number of individuals responding per age group are as follows: “21–25” *n* = 37; “26–30” *n* = 147; “31–35” *n* = 113; “36–40” *n* = 67; “41–45” *n* = 46; “46‐5 *n*=34; “51+” *n* = 59.

The influence of age on interest and support is summarized in Figure 4. Interest in the degree and support for recent genetic counseling graduates trended higher among younger participants. However, support for the degree for GCs with several years of experience was above 60% for all age groups.

### 3.5 Open Ended Comments

Analysis of open‐ended responses (totaling 12,128 words analyzed) regarding factors influencing individual opinions of a PhD in Genetic Counseling and “any additional comments” yielded nine content categories from answers provided by the 36.2% (*n* = 186/514) of participants who completed this question. Content categories included: (1) PhD for research not clinical practice, (2) Allied field alignment, (3) GCs with PhD in other fields expand reach for genetic counseling, (4) Uncertainty, (5) Funding, (6) Negative impact on MS trained GCs, (7) Equity concerns, (8) Hard no, and (9) Legacy exemption. Several responses indicated support for a PhD for GCs with specific career goals, for consistency with other allied health fields, and to enhance career opportunities and respect within the field.

1. PhD for research, not clinical practice

With respect to PhD for research not clinical practice, participants articulated that they support a PhD for GCs interested in pursuing research and/or teaching or directing as their career goals, explaining “I don't want to limit the field and think a PhD could be helpful, especially for those interested in research” (Participant 357) and “to me, the value and focus of a PhD should be on the research part of our field” (Participant 237).

2 Alignment with allied fields

Regarding alignment with allied fields, participants expressed “I support a PhD or a clinical doctorate but would love to see a doctoral degree option for GCs to advance their education/training and professional opportunities” (Participant 117) and “All other “allied healthcare professionals” have an opportunity to advance their career via a doctoral level degree” (Participant 167).

3 GCs with PhD in other fields expand reach

Multiple participants described that enhancing skills in a related field could complement the skill sets of GCs and bring additional expertize into the field. “I feel that it would be more practical to combine a master's in clinical genetic counseling with a PhD in something else to help with cross‐disciplinary research” (Participant 281).

4 Uncertainty

Many respondents expressed being conflicted about their feelings pertaining to this topic and shared feeling uncertainty about weighing the potential benefits and concerns. For example, “Honestly, I am actually conflicted on this topic, as many GCs I know are. However, there was not ‘unsure’ option for the last four questions Participant 416” and “I'm unclear as to what a PhD would add to our current roles. Many GCs already participate in research and education, as well as a variety of other non‐traditional roles” (Participant 408).

5 Additional Concerns

Many respondents had very specific concerns about funding for programs (5), negative impacts on MS trained GCs (6), a negative impact on equity in the field (7), a very clear hard no (8), and an emphasis on legacy exemption for current GCs (9). Some comments included concerns about funding and equity “GC school is pricey the way it is. Four years+ of tuition would be devastating to most. Also, DEIJ efforts would likely be affected, as those pursuing PhDs usually come from highly educated households” (Participant 170).

Additionally, there was concern about the impact on master's level GCs such as “I do worry that adding a PhD option to genetic counseling will make master's level training inadequate, similar to what happened with physical therapists” (Participant 28). Others reported concerns about faculty appointments for master level GCs “I would not be happy if places that give faculty appointments because master's is the ‘terminal degree’ then decline to do so if someone does not have a PhD” (Participant 135). Some participants expressed a resounding “No” to the idea of creating a PhD in genetic counseling “This has been researched in the past, including learning from other professions…. It would cause more problems than it solves” (Participant 184) and “If it were made available or a requirement, I'm out. Only because the MS is the terminal degree am I and several fellow GCs here and at other institutions able to hold faculty status. What a big ‘screw you’ to all MS‐level faculty counselors when a PhD comes along to remove all of their faculty appointments and potentially leadership positions, e.g., education program leadership, clinical services leadership” (Participant 58). Many pointed out concerns about what would happen to currently practicing GCs if an advanced degree became required: “Careful thought should be put into those who might need to be grandfathered in” (Participant 109). The authors acknowledge that the term “grandfathered” has been replaced by *legacy exemption* in recognition of its historical origins and associations with the former term. Of note, many who explained why they support a PhD in GC expanded their answer to also express concerns about the impact this could have on clinical masters trained GCs.

Finally, there were some comments indicating general confusion about the differences between a PhD and a clinical doctorate, and statements in which one participant made points both for and against a PhD and/or a ClinD. These types of responses emphasize the importance of clear communication about the definitions and expected outcomes of potentially new advanced degrees in the field.

## 4 Discussion

The results of this study demonstrate both meaningful interest in and significant concerns about the development of a PhD in Genetic Counseling. Over a third of participants (39.6%) reported interest in pursuing the degree themselves, suggesting a potential pool of candidates. Additionally, many indicated that reduced patient contact would not influence their decision to pursue a PhD, reflecting openness to roles beyond traditional clinical practice. However, those interested in pursuing a PhD remained in the minority, and overall support for the degree was mixed. The substantial number of open‐ended responses further underscores the importance of this topic to those in the field, with participants expressing a range of perspectives that reflect both enthusiasm for the degree and concerns about its potential impact.

Interest in a PhD and support for its development varied by age and career stage. Respondents age 21–30 were more likely to express personal interest in pursuing a doctorate (42.4%, *n* = 78/184) and to support pathways designed for recent graduates to obtain the degree (62.1%, *n* = 113/182) compared to those age 41–50 (28.8%, *n* = 23/80 and 50.0%, *n* = 39/78, respectively). More than 60% of respondents across all age groups supported a PhD pathway for GCs with several years of experience, suggesting that most older respondents still value its availability as an option for others. Significantly more respondents supported the degree for those with several years of experience than for recent graduates (69.1% vs. 57.3%; McNemar test *p* < 0.0001). This pattern may suggest a view that experience prior to pursuing a PhD may better inform real‐world questions and needs of genetic counseling research, a perspective previously reported as a perceived barrier for nurses (BSNs) considering pursuing a PhD (Granner and Ayoola 2021; Greene et al. 2017).

One of the primary themes that emerges from this study is the question of how to expand professional opportunities for genetic counselors without disrupting a predominantly master's‐trained workforce. Respondents identified several benefits and concerns consistent with prior literature (Gaupman et al. 1991; Nagy et al. 2015; Reiser et al. 2015; Walker et al. 1990). Perceived advantages included improved research and teaching skills, better access to faculty appointments and additional career opportunities, and increased professional recognition. These were balanced by concerns about the possible consequences, such as reducing opportunities for master's‐trained GCs, requiring GCs with PhDs to obtain grants to fund their work, and possibly further limiting diversity in the field. Many also noted in the open‐text responses that a PhD should not be necessary for clinical practice, consistent with prior findings (Atzinger et al. 2007). These concerns are particularly relevant given that interest in pursuing a PhD was reported by a minority of respondents (39.6%), raising questions about whether the benefits of the development of a PhD for a relatively small subset of the profession outweigh the potential harm to master's‐trained GCs. Our findings suggest that, should a PhD be more widely implemented, careful attention will be needed to ensure that roles for PhD‐trained GCs complement rather than restrict opportunities for master's‐trained individuals, as previous studies have suggested (Atzinger et al. 2007).

Some of the concerns about the potential impact on master's‐trained GCs were closely tied to the ability to maintain or achieve faculty positions for those without a PhD. This issue is nuanced, as faculty appointment requirements are not uniform across institutions and can vary across different tracks, including clinical, teaching, and research‐focused roles. Many institutions do not explicitly require a doctoral degree for faculty appointment or promotion, especially for those in clinical or teaching roles. In contrast, research‐focused and tenure‐track appointments generally require a doctoral degree to secure funding. For those on professor tracks, GCs are often evaluated using the same criteria as other faculty, regardless of degree (Wolfe Schneider 2022). Some academic institutions require faculty to hold a terminal degree in their field, allowing many genetic counseling training program faculty to be GCs with a master's degree. However, other institutions have strict criteria for faculty appointments and do not allow individuals without a doctoral level degree (e.g., a PhD or MD) to receive this title. An informal review of program websites reveals that doctoral degrees, including PhDs, EdDs, and DrPHs, are more common among program leadership than the general genetic counseling community. This may be due to a preference for advanced degrees for faculty appointments and director roles at some institutions. While a PhD in Genetic Counseling may allow easier access to faculty status which may not be available to master's‐trained genetic counselors in some institutions, there is a concern that if a PhD in Genetic Counseling becomes available, the master's degree will no longer be the terminal degree and those previously qualifying for faculty status at their institution with a master's degree may be at risk of losing that status. Further investigation of this question is warranted.

Beyond professional concerns, respondents also emphasized practical barriers to consider. The time required to pursue a PhD was the most frequently cited factor influencing personal interest, and cost was a concern for over 75%. Many genetic counseling students already incur considerable debt to fund their master's degrees, and the need to finance additional years of study would represent a significant financial barrier. Consistent with these concerns, more than half of respondents indicated a preference for online or hybrid, part‐time programs that would allow GCs to maintain their current positions. Most participants (63.8%) preferred online coursework, and almost half stated that the availability of an online learning option would be very influential in their decision to pursue an advanced degree. In contrast, very few (less than 10%) preferred traditional full‐time classes. These preferences likely reflect that many GCs who have been out of school and employed may find it challenging to move to a new location to engage in a traditional, on‐site, full‐time PhD. Respondents also raised concerns that the time and financial demands of a PhD could further limit access for individuals from historically underserved backgrounds, potentially exacerbating existing disparities within the profession. Taken together, these findings suggest that if additional PhD programs in Genetic Counseling are developed, accessibility will be a critical consideration. Potential programs could consider identifying funding sources, such as stipends and tuition remission for full‐time students, as well as flexible formats such as online, hybrid, or part‐time evening options, which may help to reduce barriers.

Despite many concerns about a PhD in Genetic Counseling, more than half of respondents were interested in pursuing a career in research (57.1%), and many reported being involved in research (55.6%). According to the 2024 NSGC PSS, GCs contribute to a variety of research activities, including involvement in student research projects, serving as research collaborators, contributing to manuscripts, presenting research at conferences, working on IRB protocols, and working as a principal investigator or co‐investigator on projects. Although many GCs feel a responsibility to share their research findings with the public, over half (53.2%) had never done so (Blanche et al. 2024). While some participants expressed concern that GCs with a PhD would need to obtain grants to fund their work, PhD programs provide training to develop advanced skills in study design, grant writing, and dissemination. Some participants voiced concerns that PhD‐trained GCs may decrease research opportunities for master's‐trained GCs. An alternative likely scenario would be increased opportunities for master's‐trained GCs, able to work on research studies run by PhD‐trained GCs who recognize and value the unique insight and research skills of their master's‐trained colleagues. While all GCs engaged in research training in their master's programs, some with rigorous research demands, advanced research training may benefit both individual career development and the broader GC and research community.

Open‐ended responses further emphasized the importance of clearly defining the difference between a PhD and a ClinD, reinforcing previous findings that further educational efforts are needed for both GCs and employers on this topic (Nagy et al. 2015; Valverde et al. 2016). Additionally, the purpose and structure of a PhD in genetic counseling need to be defined. Respondents highlighted the need to clarify program goals, including whether training would focus on research skills, counseling expertise, education, or other topics. There should be clear expectations in place that a PhD in Genetic Counseling would not change the standard for practicing master's‐trained clinical GCs to avoid a tiered system of clinicians and would not become the entry‐level degree as has been proposed for the ClinD. For GCs who do decide to pursue a PhD, obtaining a PhD in a related field, for example, genomics, public health, ethics, bioinformatics, or education, could complement the skill sets of GCs and bring additional expertize into the field that could increase recognition of GCs as important contributors to research. Alternatively, PhD in Genetic Counseling programs could offer a wide range of genetic counseling related subdisciplines including but not limited to ethics, equitable access, decision making, alternative models of care delivery, and health implementation science, expanding GCs' roles as leaders in cross‐disciplinary research. The wide range of topics of interest for a PhD is reflected in the survey results, both in the fields listed for GCs who have a PhD and the fields of interest listed for those reporting they would consider getting a PhD.

These considerations are particularly relevant as we enter an era of precision genetic therapies, where longitudinal outcomes for many rare diseases remain unknown. GCs are uniquely trained to analyze natural history data on rare diseases, interpret the impact of genetic variants, and research the psychosocial impact and outcomes of new treatments for rare diseases. Many studies support the importance of workplace and diversity in human genomics research (Gomez and Bernet 2019; Hindorff et al. 2018), and this should include a role for GCs and their unique perspectives at the forefront of genomic research. While our survey results suggest interest in developing a PhD in Genetic Counseling, significant concerns about the potential impact on master's‐level GCs necessitate careful consideration of the possible benefits and harms of developing a PhD in Genetic Counseling at the present time. Additionally, these data were collected prior to Federal shifts in research funding in the United States, which are currently having a significant downstream impact on universities. Although Federal and institutional interest in supporting GCs leading research has steadily increased in recent years, additional research is necessary to assess the impact of recent changes on enthusiasm and/or feasibility for developing a PhD in Genetic Counseling.

Alternative research training for GCs beyond the master's' degree has included certificate programs, fellowship programs, and funded research training programs, often with limited enrollment. It will be important to follow the career paths and research outcomes of those who have participated in these programs as alternative training paths to evaluate their effectiveness. Finally, since the completion of this study, two universities are offering a PhD in Genetic Counseling. Outcomes from these programs are still a few years away, and it will be critical to assess the impact on the graduates' professional opportunities as ever‐expanding roles for GCs in research and academia continue to evolve.

## 5 Limitations

Respondents working outside of academic medical centers were underrepresented compared to national data, and individuals with strong opinions, both pro and con, may have been more likely to respond, making our data less representative of the profession as a whole. Additionally, the proportion of respondents holding doctoral degrees (5.6%) and those involved in research (55.6%) was higher than expected. Both represent likely ascertainment bias, as these individuals may have a bias in favor of doctoral degrees.

The initial study title on the survey included the language “development of a doctorate degree”, but the survey introduction focused only on PhD programs referencing other professions (OT, PT, audiology) that transitioned to a PhD. Additionally, the survey did not clearly define the different types of doctoral degrees, including a ClinD, and did not propose a framework for a PhD program, which may have limited respondents' ability to assess their personal level of interest. The survey was available from November 2022 to January 2023, before the UBC PhD in Genetic Counseling program was available, possibly influencing results. Many respondents expressed confusion about whether a PhD program would be required for all GCs rather than an additional degree for those focused on research or academia, and the differences between a ClinD and a PhD, which were not defined in the survey. Finally, the survey did not ask respondents whether they supported the PhD in Genetic Counseling in general, but rather whether they supported it for recent graduates or GCs with experience.

## 6 Future Research

Further exploration of a PhD in Genetic Counseling is warranted. Given the number of GCs with doctoral degrees in adjacent fields, exploring the actual impact of having a PhD may be beneficial. Future work must also be done to (1) evaluate the potential impact on current employment, particularly for those with faculty appointments, (2) discuss the curricular content for a doctorate program, (3) identify academic institutions that could offer this degree, and (4) explore sources of funding for tuition remission and stipends. Lastly, given the limitations of this study, sampling a broader population with adequate representation of different participant demographics, practice settings, and years of experience is needed.

## Author Contributions

Elizabeth Coffran: conceptualization, data curation, formal analysis, investigation, visualization, writing original draft; Tiffiney R. Hartman: formal analysis, investigation, visualization, validation, writing review and editing; Anna Madden: writing review and editing; Ashika Mani: formal analysis, validation; Laura Conway: conceptualization, formal analysis, investigation, writing original draft, supervision; Kathleen D. Valverde: conceptualization, formal analysis, writing review, editing, supervision, and funding acquisition.

## Funding

This study and support for KDV and TRH were funded by a “Career Ladder Education Program for Genetic Counselors” grant from the Warren Alpert Foundation, Kathleen D. Valverde, PI. Grant # 10089501‐01.

## Disclosure

AI Disclosure Statement: AI was not used to draft or edit the manuscript, or in the research methods.

## Ethics Statement

All survey participants consented to the research study as the first step to completing the REDCap survey.

## Consent

Consent information included a summary of the research purpose, the length of the survey, risks and benefits, how personal information would be protected, future use of data, and contact information for questions. Participants could request a copy of the consent form by email.

## Conflicts of Interest

The authors declare no conflicts of interest.

## Supporting information


**Data S1:** The development of a doctorate degree in genetic counseling: a national survey of the opinions and concerns of genetic counselors.
