# Supplementary material for: Exploring Current Perspectives on a Doctor of Philosophy Degree in Genetic Counseling
Source: J Genet Couns. 2026 Aug 17;35(4):e70276. doi: 10.1002/jgc4.70276 (PMC13479033; doi:10.1002/jgc4.70276)
Supplement: Supplementary file 1 [file JGC4-35-0-s001.docx]

**SUPPLEMENTAL MATERIAL**

**The Development of a Doctorate Degree in Genetic counseling:**

**A National Survey of the Opinions and Concerns of Genetic Counselors**

Online Survey

PhD programs require original research and dissertations, and focus on skills that will aid graduates in a career in research or teaching at an advanced level. Many other health care fields have already transitioned to offering a PhD, including audiology, pharmacy, nursing, physical therapy, and occupational therapy. This survey will be asking about your thoughts on the creation of a PhD program with a focus on genetic counseling.

Demographics:

1. Age. Open text
2. Year of Graduation from Genetic Counseling Masters Program. Open text
3. Number of years you have worked as a Genetic Counselor. Open text
4. Are you currently working as a Genetic Counselor? Y/N
5. What region are you currently practicing in?

a. Region 1 (CT, MA, ME, NH, RI, VT, CN, maritime provinces)

b. Region 2 (DC, DE, MD, NJ, NY, PA, VA, WV, PR, VI, Quebec)

c. Region 3 (AL, FL, GA, KY, LA, MS, NC, SC, TN)

d. Region 4 (AR, IA, IL, IN, KS, MI, MN, MO, NE, ND, OH, OK, SD, WI, Ontario)

e. Region 5 (AZ, CO, MT, NM, TX, UT, WY, Alberta, Manitoba, Saskatchewan)

f. Region 6 (AK, CA, HI, ID, NV, OR, WA, British Columbia)

6. What is your job setting?

a. Academic hospital/Medical facility

b. Non-academic hospital / medical facility

c. Private practice or physician private practice

d. Non-profit or government organization

e. Diagnostic Laboratory

f. Other company

g. University, college, or training program

h. Other (text for other answers)

7. Sex/gender

a. male

b. female

c. transgender

d. nonbinary

e. prefer not to say

8. Race

a. American Indian, Alaskan Native, or Indigenous Peoples of Canada

b. Asian

c. Black, African American, or of African descent

d. Middle Eastern or North African

e. Native Hawaiian or other Pacific Islander

f. White

9. Ethnicity

a. Hispanic or Latino/Latina

b. Not Hispanic or Latino/Latina

Education:

10. What master’s degrees have you earned?

a. MS/MA/ScM/MSc/MGC Genetic Counseling

b. MS/MA/ScM/MSc/MGC Genetics (Human/Medical)

c. MS/MA Other

d. MBA

e. MPH

f. BSN/MSN

g. MSW/MSSW

h. Med

i. Other (open text for other)

11. Separately (for skip logic), do you have a doctoral degree? Y/N

a. If yes, what type

1. PhD
2. EdD
3. DrPh
4. MD
5. JD
6. Other

A, What field is your doctoral degree in? Open text

B. Did you earn your doctorate before or after the genetic counseling degree? B/A

b. If no, did you consider applying to a dual master’s/doctoral degree for GC school? Y/N

c. Have you considered earning a doctorate since you received your MS degree? Y/N

A. If yes, which field have you considered getting a doctorate in? open text

12. [open ended] What are your career goals for the next 5 years?

13. Do you currently participate in research? Y/N

a. If yes: Please describe your current research participation. Open text

Likert scale questions:

14. Please indicate your agreement with the following statements: Agree strongly/agree/neither agree nor disagree/disagree/disagree strongly

a. Genetic counselors who have a leading role in research have more autonomy than those who do not.

b. Genetic counselors who have a leading role in research have more career opportunities than those who do not.

c. A PhD in genetic counseling will garner genetic counselors more respect

d. A PhD in genetic counseling will increase the visibility of the genetic counseling field

e. A PhD in genetic counseling will increase diversity in the field

f. A PhD in genetic counseling will allow genetic counselors to be more autonomous

g. A PhD in genetic counseling will require genetic counselors to obtain grants to fund their work

h. A PhD in genetic counseling will enhance genetic counselors’ research skills

i. A PhD in genetic counseling will provide genetic counselors with more professional opportunities

j. A PhD in genetic counseling will increase genetic counselors’ professional satisfaction

k. A PhD in genetic counseling will increase genetic counselors’ ability to get faculty positions

l. A PhD in genetic counseling will decrease opportunities for genetic counselors with a master’s degree

m. A PhD in genetic counseling will enhance genetic counselor’s teaching and educational skills

15. Would you be interested in pursuing this degree? (Yes / No)

16. Please indicate how much influence these factors would have in **your** decision to pursue a PhD in genetic counseling.

Not a factor / Somewhat influential / moderately influential / very influential

a. Cost of attendance

b. Amount of time required to pursue the degree

c. If it was required by your employer for career advancement

d. Ability to work up to 50% time while earning the degree

e. Ability to work more than 50% time while earning the degree

f. Availability of an online-learning option

g. Level of support from current employer

h. Expectation of current employer to have an advanced degree

i. Ability to apply genetic counseling coursework or experience as credit towards the advanced degree

j. Opportunity to increase salary

k. Decrease in the amount of patient contact

l. Concern about effects on licensure

17. Are there any other factors that would influence your decision to pursue an advanced degree in genetic counseling? Open text

18. Which of the following options would you prefer if you were going to pursue your doctorate in genetic counseling? Select all that apply.

a. Online classes

b. Part time night classes

c. Traditional full-time classes

d. Weekend classes

e. Hybrid – online with residency requirements – come in person a number of times a year

f. Will not pursue a doctorate

g. Other (open text)

19. Do you support the development of a PhD in genetic counseling for genetic counselors with several years of experience? Y/N

20. Do you support the development of a PhD in genetic counseling for recent MSGC graduates? Y/N

21. Are you interested in working in research? Y/N

22. Are you interested in working in teaching / academia? Y/N

23. Please provide any additional comments you may have. Open Text
